# DNA Methyltransferase Expression (DNMT1, DNMT3a, and DNMT3b) as a Potential Biomarker in Age-Related Macular Degeneration

**DOI:** 10.3390/jcm14020559

**Published:** 2025-01-16

**Authors:** Pedro Camacho, Edna Ribeiro, Bruno Pereira, João Nascimento, Paulo Caldeira Rosa, José Henriques, Sandra Barrão, Silvia Sadio, Bruno Quendera, Mariana Delgadinho, Catarina Ginete, Carina Silva, Miguel Brito

**Affiliations:** 1 H&TRC—Health & Technology Research Center, ESTeSL—Escola Superior de Tecnologia da Saúde, Instituto Politécnico de Lisboa, 1990096 Lisbon, Portugal; edna.ribeiro@estesl.ipl.pt (E.R.); bruno.pereira@estesl.ipl.pt (B.P.); mariana.delgadinho@estesl.ipl.pt (M.D.); carina.silva@estesl.ipl.pt (C.S.); miguel.brito@estesl.ipl.pt (M.B.); 2 Retina Institute of Lisbon, 1150085 Lisbon, Portugal; cirurgia@sapo.pt (J.N.); paulocaldeirarosa@gmail.com (P.C.R.); jose.henriques@retinaplus.com (J.H.); silvia.sadio@ulssjose.min-saude.pt (S.S.); 3 iNOVA4Health, NOVA Medical School, NMS, Faculdade de Ciências Médicas, FCM, Universidade NOVA de Lisboa, 1169056 Lisboa, Portugal; 4 Beatriz Ângelo Hospital, 2674514 Lisbon, Portugal; 5 Ophthalmology Department, Centro Hospitalar Universitário de Lisboa Central, 1150199 Lisbon, Portugal; sandra.barrao@gmail.com (S.B.); bruno.alexandre.pq@gmail.com (B.Q.)

**Keywords:** age-related macular degeneration, epigenetics, geographic atrophy, choroidal neovascularization, SD-OCT

## Abstract

**Background/Objectives:** Age-related macular degeneration (AMD) is a global cause of vision loss, with limited therapeutic options highlighting the need for effective biomarkers. This study aimed to characterize plasma DNA methyltransferase expression (*DNMT1*, *DNMT3A*, and *DNMT3B*) in AMD patients and explore divergent expression patterns across different stages of AMD. **Methods**: Thirty-eight AMD patients were prospectively enrolled and stratified by disease severity: eAMD, iAMD, nAMD, and aAMD. Comprehensive ophthalmological assessments were performed, including best-corrected visual acuity, digital color fundus photographs, and Spectral Domain Optical Coherence Tomography. Peripheral blood samples were collected for RNA extraction and qRT-PCR to access epigenetic effectors’ transcriptional expression, namely *DNMT1*, *DNMT3A*, and *DNMT3B* genes. The collected data were analyzed using IBM SPSS 29. **Results:** *DNMT1* expression was significantly downregulated in late AMD (−0.186 ± 0.341) compared to early/intermediate AMD (0.026 ± 0.246). Within late AMD, aAMD exhibited a marked downregulation of *DNMT1* (−0.375 ± 0.047) compared to nAMD (0.129 ± 0.392). *DNMT3A* and *DNMT3B* showed similar divergent expression patterns, correlating with disease stage. **Conclusions:** This study identified stage-specific transcriptional differences in *DNMT* expression, emphasizing its potential as a biomarker for AMD progression and a target for future research into personalized therapeutic strategies.

## 1. Introduction

With a prevalence nearly three times higher than Alzheimer’s disease, age-related macular degeneration (AMD) affects almost 200 million people worldwide, and despite some differences between countries, population aging is expected to contribute to a potential increase to nearly 300 million cases by 2040 [1].

Despite advances in neovascular AMD (nAMD) treatment, the lack of therapeutic options for non-late stages (approximately 90%) and for late atrophic stages (aAMD) [2,3] underscores the imperative to identify potential biomarkers as a public health strategy [4]. These biomarkers are essential for the study of disease progression [5] and monitoring [6] and current and future therapies [7].

The use of multimodal imaging has been pivotal in the search for potential AMD biomarkers across various stages of development and progression, including intermediate forms [2,8,9], as well as in the treatment and progression of nAMD [10,11] and aAMD [12]. Associations between dietary habits, inflammatory and immune changes, and lipid metabolism in AMD have been described [13]. Furthermore, genetic variants in complement factor H pathways, age-related maculopathy susceptibility 2/Serine protease HTRA1 (ARMS2/HTRA1) [14,15], lipid metabolism [15,16], energy pathways [17], and inflammatory (TIMP3) pathways [15] have been identified in AMD. However, genetics can only explain 40–60% of the disease [18], and their relationship with non-invasive imaging biomarkers remains limited in human studies.

Epigenetic mechanisms, particularly DNA methylation, are emerging as key modulators in AMD pathogenesis. Epigenetic modifications, which regulate gene expression in response to environmental stimuli, are reversible processes and thus present promising targets for therapeutic interventions [19,20]. Initial studies have identified altered methylation patterns in antioxidant genes such as *GSTM1* and *GSTM5* in AMD patients, suggesting links to oxidative stress pathways [21,22]. Additionally, methylation changes in the *IL-17RC* gene have been reported, and are associated with pro-inflammatory cytokines IL-17A and IL-17F and near the ARMS2 locus in nAMD [23].

Although these findings are promising, substantial gaps persist in understanding the role of epigenetics in AMD progression. Current studies often rely on post-mortem tissue samples and lack correlations with real-time clinical biomarkers [20]. Moreover, the pathways driving AMD progression from early to late stages remain unclear [21,24].

This study investigated the transcriptional expression patterns of DNA methyltransferases (*DNMT1*, *DNMT3A*, and *DNMT3B*) across different stages of AMD progression. DNMTs are responsible for maintaining DNA methylation patterns, and their altered expression has been linked to inflammation, oxidative stress, and cellular aging—key mechanisms in AMD pathogenesis [25,26]. By characterizing DNMT expression across early, intermediate, and late AMD stages, we sought to uncover potential epigenetic biomarkers that could aid disease monitoring and therapeutic development.

Given prior evidence linking age-related DNA methylation changes to lipid system dysfunction—a critical pathway for maintaining retinal pigment epithelium (RPE) and photoreceptor health [25]—and the association between lipid metabolism impairments and drusen formation, a hallmark of AMD progression [26], we hypothesized that altered DNMT expression may contribute to disease evolution. Our study provides new insights into the potential clinical relevance of DNMTs in AMD, emphasizing their role in disease monitoring and personalized treatment strategies.

## 2. Materials and Methods

### 2.1. Study Design and Setting

This prospective cross-sectional study was conducted as part of the “DNA Methyltransferase as a Potential Biomarker for Anti-VEGF Therapy in Neovascular Age-related Macular Degeneration (IPL/IDI&CA2023/DETECTnAMD_ESTESL)” project at Instituto de Retina de Lisboa (IRL) and Instituto de Oftalmologia Dr. Gama Pinto (IOGP). Ethical approval was obtained from both institutions’ Institutional Ethical Review Boards before the study’s initiation. The study adhered to the ethical principles outlined in the Declaration of Helsinki, and all participants provided informed consent after receiving a detailed explanation of the study’s objectives and their involvement before any study procedure.

### 2.2. Patient Recruitment and Classification

Thirty-eight AMD patients were prospectively enrolled during routine appointments at IRL and IOGP. Patients were classified according to the Age-Related Eye Disease Study (AREDS) [27] criteria and more recent clinical classification [28]. Only patients with complete ophthalmological data, including best corrected visual acuity (BCVA), digital color fundus photographs (CFP) Topcon (Topcon DRI OCT Triton; Topcon, Corp., Tokyo, Japan), and Spectral Domain Optical Coherence Tomography (SD-OCT) imaging (Spectralis; Heidelberg Engineering, Heidelberg, Germany) obtained during the same visit, were invited to participate. Additional demographic and clinical data, including age, intraocular pressure (IOP), spherical equivalent (SE), and relevant clinical information, were also collected for each patient. The SD-OCT acquisition protocol includes a High-resolution macular volume scan (20° × 20°, 49 raster horizontal B-scans, 7 frames per scan), with 1024 A-scans per B-scan and a depth resolution of 3.9 µm.

The staging of each patient was performed blindly for clinical and demographic data. In disagreement, one of the senior investigators conducted the final classification. Patients were initially divided into non-late AMD (early AMD [eAMD] and intermediate AMD [iAMD]) and compared with late AMD (neovascular AMD [nAMD] and atrophic AMD [aAMD]). Subsequently, the groups were stratified and compared based on severity: eAMD, iAMD, nAMD, and aAMD. Clinical categorization criteria [27] and procedures [28] were as follows: eAMD (AREDS category 2) with intermediate drusen (63–124 μm in diameter) or mild RPE abnormalities; iAMD (AREDS category 3) with numerous intermediate drusens, at least one large drusen (≥125 μm in diameter), and areas of RPE atrophy not involving the center of the fovea; late atrophic AMD (AREDS category 4) with geographic atrophy in the central subfield (inside 500 µm radius from foveal center); and late neovascular AMD (AREDS category 4) with fibrovascular/serous pigment epithelial detachment, serous (or hemorrhagic) sensory retinal detachment, subretinal/sub-retinal pigment epithelial hemorrhage, or subretinal fibrous tissue (or fibrin) related to neovascular AMD.

Inclusion Criteria: Patients aged 55 years or older with a confirmed medical diagnosis of AMD (early, intermediate, neovascular AMD, and atrophic AMD) and multimodal assessment performed on the day of blood collection.

Exclusion Criteria: Patients with any ocular diagnosis that may confound the study outcome (such as diabetic retinopathy, hereditary retinal dystrophies, or glaucoma), high myopia or hyperopia (greater than 6 diopters spherical equivalent), media opacification, ocular inflammation, history of retinal detachment, photodynamic therapy, or intravitreal injection (including triamcinolone) within 90 days of the study. Additionally, patients with a history of cancer, autoimmune disorders, dementia, and/or other neurological disorders were excluded.

### 2.3. Sample Collection and Epigenetic Profile

During the routine consultation, a 1 mL peripheral blood sample was collected, and the total RNA was extracted using Quick-RNA™ Whole Blood (Zymo Research, Orange, CA, USA), according to the manufacturer’s instructions. The concentration and purity of all RNA samples were determined on a NanoDrop One spectrophotometer (Thermo Scientific, Waltham, MA, USA). One-step NZY Speedy RT-qPCR Green kit (NZYtech, Lisbon, Portugal) was used for first-strand cDNA synthesis and subsequent quantitative real-time PCR in a final volume of 20 μL and performed on the CFX Connect™ Real-Time PCR Detection System (Bio-Rad, Hercules, CA, USA) to quantify gene expression. Each reaction took place in triplicate using in every reaction non-template control and specific primers, listed in Table 1, for the genes *DNMT1*, *DNMT3A, DNMT3B*, and a reference gene, *GAPDH*, which was used for data normalization. The cycling conditions were as follows: 50 °C for 15 min, 95 °C for 5 min, and 40 cycles of 95 °C for 15 s and 60 °C for 45 s with fluorescent readings. Then, the relative quantification of the target genes was undertaken by normalizing threshold cycles (Ct) with the mean Ct of GAPDH. Transcript levels were analyzed by calculating ΔΔCt (ΔΔCt = ΔCt treatment—average ΔCt control), and the obtained ΔΔCt values were subsequently log 2-transformed for graphical purposes.

### 2.4. Workflow Overview

**Figure 1:**
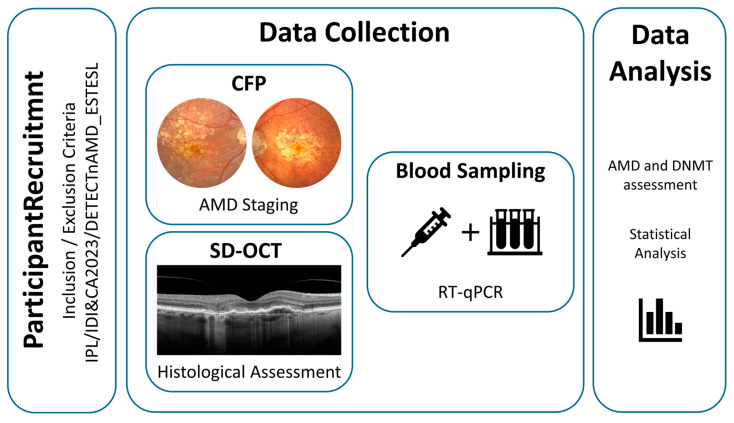
Schematic representation of the study. Example of two-color fundus photography images of iAMD and Late AMD (atrophic stage) participants; Right eye SD-OCT examination disclosing an area of perifoveal complete outer retinal pigment and outer retinal atrophy (cRORA) along with a shallow irregular RPE detachment associated with subretinal fluid and subretinal hyperreflective material, which is compatible with type 1 macular neovascularization; Example of peripheral blood collection for epigenetic profiling.

A schematic representation of the study workflow is provided in Figure 1 to complement the detailed description above. The diagram outlines the key steps, including participant recruitment, data collection, and assessment of AMD and *DNMTs*.

### 2.5. Statistical Analysis

The collected data were analyzed using Statistical Package for the Social Sciences IBM SPSS (Version 29.0) software. Descriptive statistics were used to characterize the study data, including absolute frequencies, relative frequencies, and mean values with standard deviations. To compare two independent groups *t*-tests were used after verifying normality through the Shapiro–Wilk test, or the non-parametric Mann–Whitney test when normality was not verified. Differences between all AMD stage groups were evaluated using one-way ANOVA or the non-parametric Kruskal–Wallis test whenever normality and/or homoscedasticity were not verified, and Tukey’s test was used for multiple comparisons. A chi-squared test was used to assess group homogeneity when comparing qualitative characteristics. A significance level of 5% was considered for all analyses.

## 3. Results

In this work, we accessed and described the clinical profile of the 38 AMD patients enrolled, as summarized in Table 2. Patients were initially categorized into early/intermediate AMD (33.3% male and 66.7% female) and late AMD (55.5% male and 45.5% female), with mean ages of 81.6 ± 3.8 years and 83.9 ± 6.0 years, respectively. While age is slightly increased in the late AMD group, this difference was not statistically significant (*p* = 0.105).

The most notable difference between groups was observed in BCVA. As expected, patients in the early/intermediate AMD group had significantly better visual function, with a mean BCVA of 74.7 ± 8.6 letters compared to 49.9 ± 20.7 letters in the late AMD group (*p* < 0.001). This finding reflects the progressive visual decline characteristic of late AMD stages.

Additional parameters such as IOP and SE were comparable between groups, with no statistically significant differences (IOP: *p* = 0.718; SE: *p* = 0.992). Similarly, retinal thickness measures, including central retinal thickness (CRT) and central foveal thickness (CFT), did not differ significantly between early/intermediate and late AMD groups (CRT: *p* = 0.874; CFT: *p* = 0.443).

Regarding systemic conditions, hypertension was slightly more prevalent in the late AMD group (65.7%) than in the early/intermediate AMD group (61.1%). In comparison, diabetes mellitus (DM) was more common in the early/intermediate AMD group (44.4%) than in the late AMD group (30%). However, neither difference reached statistical significance (hypertension: *p* = 0.804; DM: *p* = 0.357).

**Figure 2:**
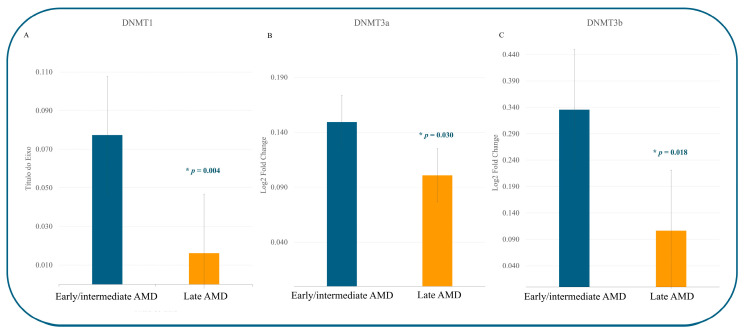
Data represents the relative expression of DNA methyltransferase genes between early/intermediate AMD and Late AMD. (**A**) *DNMT1* expression; (**B**) *DNMT3a* expression; and (**C**) *DNMT3b* expression. GAPDH was used for normalization (ΔΔCt values were log-transformed). Significant *p*-values, which were calculated using a *t*-test, are denoted by *.

Figure 2 illustrates the relative expression levels of DNA methyltransferase genes (*DNMT1*, *DNMT3A*, and *DNMT3B*) in early/intermediate AMD compared to late AMD. The transcriptional expression analysis reveals distinct patterns of expression associated with different AMD stages. In Figure 2A, a significant downregulation of *DNMT1* expression is evident in late AMD (−0.186 ± 0.341) compared to early/intermediate AMD (0.026 ± 0.246; *p* = 0.004).

Additionally, among the de novo DNA methyltransferases, *DNMT3A* and *DNMT3B* exhibited significant downregulation in late AMD. Specifically, *DNMT3A* expression was reduced to −0.223 ± 0.456 in late AMD versus 0.044 ± 0.364 in early/intermediate AMD (*p* = 0.030). Similarly, *DNMT3B* expression decreased to −0.400 ± 0.569 in late AMD compared to 0.057 ± 0.550 in early/intermediate AMD (*p* = 0.018).

These findings highlight the stage-specific transcriptional alterations in *DNMT* expression, underscoring their potential involvement in AMD progression.

In phase 2 (Table 3), patients were stratified into four groups based on AMD severity: eAMD, iAMD, nAMD, and aAMD. While the sample sizes were unbalanced across groups, no significant differences were observed in mean age (*p* = 0.164), intraocular pressure (IOP; *p* = 0.547), or spherical equivalent (SE; *p* = 0.650) among the groups.

Significant differences, however, were noted in BCVA (*p* < 0.001). As expected, patients with eAMD and iAMD demonstrated the highest BCVA values, with mean ETDRS letter scores of 77 ± 9.9 and 74.4 ± 8.8, respectively. In contrast, BCVA was markedly reduced in patients with aAMD (44.7 ± 14.7) and nAMD (44.8 ± 23.3), reflecting the advanced disease stages and their associated impact on visual function.

Although CRT did not reach statistical significance (*p* = 0.059), patients with nAMD exhibited the highest CRT (332.9 ± 132.7 µm), notably more significant than in other groups. Conversely, the lowest CRT values were observed in aAMD patients (222.3 ± 51.3 µm), consistent with the retinal thinning typically associated with atrophic changes. Similarly, CFT showed no significant differences across groups (*p* = 0.354), but the lowest values were seen in aAMD patients (172 ± 79.3 µm), reflecting structural degeneration at the fovea.

These findings highlight the progressive impact of AMD on visual acuity and retinal morphology, with distinct patterns observed between atrophic and neovascular forms. While CRT and CFT did not show statistically significant differences, the observed trends align with the clinical characteristics of the disease stages.

**Figure 3:**
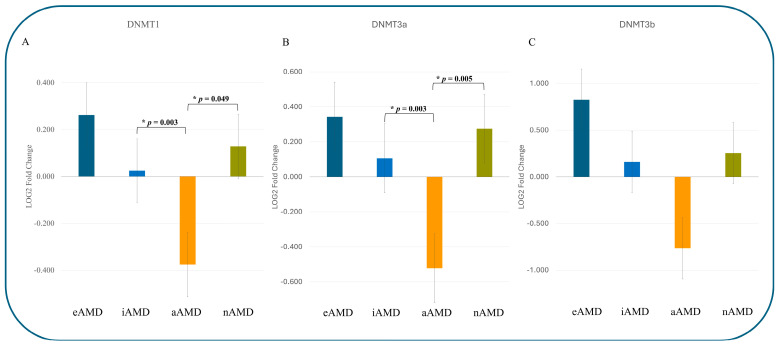
Data represent the relative expression of DNA methyltransferase genes across all AMD stages. (**A**) *DNMT1* expression; (**B**) *DNMT3a* expression; and (**C**) *DNMT3b* expression. GAPDH was used for normalization (ΔΔCt values were log-transformed). Significant *p*-values, calculated by Tukey’s test, are denoted by *.

Interestingly, in phase 2, divergent expression patterns were observed in the transcription of DNA methyltransferase genes (*DNMT1*, *DNMT3A*, and *DNMT3B*) across all AMD stages, as shown in Figure 3. Data reveal significant downregulation of *DNMT1* expression (*p* = 0.003) in aAMD (−0.375 ± 0.047) compared to intermediate AMD (0.025 ± 0.2043) (Figure 2A). Significant downregulation of *DNMT1* expression was detected in aAMD (−0.375 ± 0.047) compared to intermediate AMD (0.025 ± 0.2043; *p* = 0.003) and nAMD (0.129 ± 0.392; *p* = 0.049) (Figure 2A).

For de novo DNA methyltransferases, a notable downregulation of *DNMT3A* expression was observed in aAMD (−0.522 ± 0.064) compared to intermediate AMD (0.106 ± 0.316; *p* = 0.003). Additionally, differences in *DNMT3A* expression were evident among the late AMD groups (*p* = 0.005), with a significant upregulation in nAMD (0.275 ± 0.467).

Although *DNMT3B* expression did not show statistically significant differences, a similar trend of downregulation in aAMD and upregulation in nAMD was clinically observed. These findings underscore distinct transcriptional profiles of DNA methyltransferase genes across AMD stages, with potential implications for disease-specific epigenetic regulation.

## 4. Discussion

While genetic variants in different pathways such as complement cascade [14,15], lipid metabolism [15,16], energy production [17], and inflammation [15] have been identified in AMD, genetics alone do not fully explain the etiology and progression of the disease, with only 40–60% of cases accounted for [18]. This partial genetic explanation [29] becomes evident in studies of twins with discordant AMD phenotypes, highlighting the significant role of non-genetic factors, including epigenetic modifications, in AMD pathogenesis [30,31].

### 4.1. DNA Methyltransferase Expression in Patients with Early/Intermediate AMD vs. Late AMD

Herein we aimed to explore the expression of plasma DNA methyltransferases (*DNMT1*, *DNMT3A*, and *DNMT3B*) in individuals with early/intermediate AMD compared to those with late-stage AMD.

Our group recently demonstrated the viability of peripheral blood samples as surrogate tissue, highlighting their potential for minimally invasive epigenetic profiling in retinal disease [32].

Given the close association between age, RPE changes, and related epigenetic patterns in AMD [33,34,35,36], it is essential to highlight that our analysis revealed no significant differences in age between the early/intermediate AMD and late AMD groups. While previous studies have reported inconsistent findings regarding sex-related differences in AMD prevalence, no statistically significant sex differences were observed in our study groups. Nonetheless, it is essential to recognize that both sex and age may influence complement system activity in patients with iAMD [37].

As expected, significant disparities in visual function were observed between the groups (*p* < 0.001), with late AMD patients exhibiting notably lower BCVA compared to those with early/intermediate AMD. This finding aligns with the known progression of AMD severity.

Traditionally, studies tend to compare early/intermediate AMD with late AMD. However, some caution is needed when interpreting results due to the heterogeneous nature of late AMD, which includes distinct phenotypes such as nAMD with retinal thickening and aAMD with retinal and RPE thinning. For instance, although differences in retinal metrics were anticipated, our analysis revealed no statistically significant differences in retinal thickness between early/intermediate and late AMD. However, a closer examination revealed that the standard deviation in late AMD (124 µm) is nearly three times higher than that in early/intermediate AMD (38.3 µm). This variability likely reflects the presence of both choroidal neovascularization and atrophic lesions in the late AMD group.

Building upon this conventional approach, we investigated DNA methyltransferase (*DNMT*) expression between early/intermediate AMD and late AMD. Our data unveiled a downregulation of *DNMT1* expression in late AMD compared to early/intermediate AMD (*p* = 0.004).

Previous studies have linked reduced *DNMT1* expression to inflammatory conditions and hypomethylation of IL17RC, a gene implicated in chronic inflammation in AMD [38], which aligns with our findings [29]. Additionally, oxidative stress [39], a hallmark of AMD, has been associated with downregulation patterns in de novo *DNMT*s [38]. Consistent with this, our study showed significant downregulation of *DNMT3A* (*p* = 0.030) and *DNMT3B* (*p* = 0.018) in late AMD.

Interestingly, the increased expression of *DNMT*s observed in early/intermediate AMD may reflect early disease changes potentially linked to aging rod photoreceptors. These early-stage changes have been associated with hypermethylation of antioxidant gene promoters such as *GSTM2, GSTM5*, and *GSTM6* [22,36]. Despite the complex DNA methylation modulation observed in AMD progression and severity, elucidating these intricate interactions and their implications remains challenging for future research [34,40]. Notably, understanding the differential patterns of *DNMT*s [34], such as hypomethylation at the ARMS2/HTRA1 locus or hypermethylation at the protease serine 50 (PRSS50) locus, is pivotal in delineating AMD pathogenesis [23]. These epigenetic changes may serve as key insights into the molecular mechanisms underlying AMD and as potential biomarkers for disease stratification.

In an era characterized by diverse therapeutic options for nAMD and emerging treatments for aAMD, identifying robust biomarkers capable of distinguishing phenotypes with distinct underlying mechanisms is crucial. Such biomarkers would facilitate personalized treatment strategies, aligning with the principles of precision medicine and improving AMD management outcomes [23,39].

### 4.2. DNA Methyltransferase Expression Across Different AMD Stages

Early investigations focused on AMD epigenetic modifications suggested potential *DNMT* abnormalities linked to oxidative stress [22]. However, many of these studies relied on post-mortem retinal pigment epithelium (RPE)/choroid samples and lacked precise differentiation among AMD patients, potentially leading to inconsistent findings [23].

In this context, the second phase of our study, although exploratory with a sample size similar to others [41], presents findings on DNA methyltransferase expression across different stages of AMD.

Stratification of the sample by AMD stage resulted in slight group imbalances: eAMD (2 patients), iAMD (16 patients), aAMD (6 patients), and nAMD (14 patients). Significant differences were observed in BCVA (*p* < 0.001), reflecting different severity stages, with early and intermediate AMD groups exhibiting the highest ETDRS letter values (77 ± 9.9 letters and 74.4 ± 8.8 letters, respectively), as expected.

While no significant differences were found in CRT (*p* = 0.059) and foveal thickness (*p* = 0.354), clinically evident differences in AMD retinal metrics were observed. Patients with aAMD showed the lowest thickness values (CRT 222.3 µm; CFT 172 µm), whereas those with nAMD exhibited the highest values (CRT 332.9 µm; CFT 217 µm).

Despite the relatively small sample size, intriguing findings emerged regarding *DNMT* expression. Compared to early/intermediate AMD versus late AMD, phase 2 analysis revealed differential *DNMT* expression between aAMD and nAMD, suggesting distinct methylation patterns in late-stage disease. Recent studies have described numerous hypermethylated and hypomethylated fragments in AMD, underscoring the complexity of methylation alterations [42].

Our results illustrate the variety of mechanisms at play in late AMD. A significant downregulation of *DNMT1* expression was observed in aAMD compared to both iAMD (*p* = 0.003) and nAMD (*p* = 0.049). This downregulation may be related to complement cascade upregulation, which endorses a significant role in AMD pathogenesis [43]. Additionally, decreased expression of SIRT1 and Oct4 in aged retinas and RPE cells of AMD patients [33] suggests an increased susceptibility to apoptosis, oxidative stress, and inflammatory damage [33,44].

In contrast, an upregulation of *DNMT1* expression was noted in nAMD compared to aAMD (*p* = 0.049). This increased expression in nAMD tissues, coupled with the upregulation of both *DNMT1* and *DNMT3B* in the nAMD group, mirrors findings from experimental models of induced nAMD. In particular, this mouse model study demonstrated a concurrent downregulation of Notum, a secretion inhibitor of Wnt signaling, in the RPE/choroidal complexes [42]. Given the predominant involvement of the oxidative stress pathway [38] and the role of complement factors, downregulation in de novo *DNMT*s may have been anticipated. A significant downregulation of *DNMT3A* expression (*p* = 0.003) was observed in aAMD compared to iAMD. Furthermore, disparities in *DNMT3A* expression were evident among late AMD groups (*p* = 0.005), with an upregulation observed in nAMD. Although no statistical differences were detected in *DNMT3B* expression, a consistent pattern across different AMD types was clinically observed.

### 4.3. Biological Significance of Differential DNMT Expression and Clinical Implications

Our study underscores the potential role of differential *DNMT* expression in AMD pathogenesis, which could build a potential strategy for disease monitoring and personalized treatment. The observed downregulation of *DNMT1* in aAMD, contrasted with its relative upregulation in nAMD, suggesting that distinct epigenetic mechanisms may drive disease progression. Previous studies have highlighted the critical role of standard methylation patterns in the development and homeostasis of the human retina, particularly in photoreceptors and RPE—key structures affected in AMD [45].

One key example is the methylation of the ELOVL2 (Elongation of Very Long Chain Fatty Acids-Like 2) promoter, which has been recognized as a highly predictive epigenetic marker of biological age and plays a pivotal role in retinal biology [46,47]. This gene’s methylation status impacts various retinal structures, including photoreceptors and the RPE, which are central to AMD pathophysiology. Indeed, the link between long-chain polyunsaturated fatty acids (LCPUFAs) and AMD has been well-documented in the AREDS reports, further reinforcing the connection between lipid metabolism and AMD progression [47].

Our findings appear to reflect a broader interplay between key biological processes implicated in AMD, such as oxidative stress [38], complement activation, and chronic inflammation. These processes are known contributors to retinal damage, and our observation of altered *DNMT3A* and *DNMT3B* expression across different AMD stages could support their role in regulating cellular metabolism, DNA repair mechanisms, and immune responses. Alterations in LCPUFAs have been shown to modulate immune activity and inflammatory pathways, potentially driving the development of choroidal neovascularization—one of the hallmark features of late AMD [25,47,48].

Additionally, studies have suggested the importance of ELOVL2 methylation in AMD, linking its dysfunction to early sub-RPE deposits, which share similarities with drusen, a key biomarker of AMD progression [48]. The loss of ELOVL2 function may disrupt lipid metabolism and initiate pathological changes in the retina, thereby contributing to disease progression.

Together, these patterns highlight the critical role of *DNMT*s as mediators of epigenetic regulation in AMD.

Clinically, these findings offer promising implications for developing minimally invasive biomarkers. Peripheral blood profiling of *DNMT* expression could be a practical tool for monitoring disease progression and stratifying AMD phenotypes. For instance, the observed *DNMT* downregulation in aAMD and its distinct expression in nAMD highlight the potential to distinguish late-stage phenotypes. Such biomarkers could enhance early diagnosis, inform prognosis, and guide personalized therapeutic interventions.

However, validation through larger, longitudinal studies is essential to establish these applications fully. Several limitations of this study must be acknowledged. The cross-sectional design restricts causal inferences about the observed patterns, while the relatively small sample size, particularly after stratification, limits the generalizability of findings. The unbalanced female-to-male ratio could also influence the results, despite the lack of observed statistical differences between sexes. Future studies with larger, more balanced cohorts are needed to validate and expand on these findings.

Nonetheless, this study’s reliance on real-world data enhances its relevance and underscores the feasibility of peripheral blood-based epigenetic profiling. Some uncontrolled variables may have influenced the results despite efforts to account for potential lifestyle factors and genetic variations.

In conclusion, this study identifies distinct *DNMT* expression patterns across AMD stages, offering valuable insights into the potential role of epigenetic regulation in AMD pathophysiology. While these findings provide a foundation for exploring *DNMT*s as biomarkers and therapeutic targets, further research is needed to validate these observations and fully elucidate their clinical significance. Future investigations should aim to build on these results to enhance our understanding of AMD progression and support the development of more precise diagnostic and therapeutic approaches.

## Figures and Tables

**Table 1:** Primer sequences, accession numbers, and product lengths for qRT-PCR analysis.

Genes	Accession Number *	Forward Primer (5′→3′)	Reverse PRIMER (3′→5′)	Product Length (bp)
*GAPDH*	NM_002046.7	GAGTCAACGGATTTGGTCGTA	GCAGAGATGATGACCCTTTTG	245
*DNMT1*	NM_001379.4	CCTCCAAAAACCCAGCCAAC	TCCAGGACCCTGGGGATTTC	101
*DNMT3A*	NM_022552.5	CCAACATCGAATCCATGAAA	CTTGCGCTTGCTGATGTAGT	140
*DNMT3B*	NM_175850.3	CGAATTTTACCACCTGCTGAATT	AGAACGGCCGGTCATCAC	59

* NCBI Reference Sequence (National Center for Biotechnology).

**Table 2:** Overall clinical characterization of the sample according to study group.

	Early/Intermediate AMD (*n* = 18)	Late AMD (*n* = 20)	*p-*Value
Age (years) Mean (SD)	81.6 (3.8)	83.9 (6.0)	0.105 ^a^
Sex M/F *n* (%)	6 (33.3%) 12 (66.7%)	11 (55.0%) 9 (45.0%)	0.169 ^b^
Study eye RE/LE *n* (%)	8 (44.4%) 10 (55.6%)	10 (50.0%) 10 (50.0%)	0.732 ^b^
BCVA Mean (SD)	74.7 (8.6)	49.9 (20.7)	<0.001 ^c^
IOP (mmHg) Mean (SD)	14.7 (2.4)	14.8 (3.1)	0.718 ^a^
SE Mean (SD)	0.26 (0.89)	0.23 (0.77)	0.992 ^a^
Hypertension Yes/no (%)	11 (61.1%) 7 (38.9%)	13 (65.7%) 7 (35.0%)	0.804 ^b^
DM Yes/no (%)	8 (44.4%) 10 (55.6%)	6 (30.0%) 14 (70.0%)	0.357 ^b^
CRT Mean (SD)	281.3 (38.3)	299.8 (124)	0.874 ^a^
CFT Mean (SD)	217.7 (27.5)	204.2 (73.8)	0.443 ^c^

*n* = absolute frequency; SD = standard deviation; M = male; F = female; RE = right eye; LE = left eye; % = proportion per group; BCVA = best corrected visual acuity; IOP = intraocular pressure; mmHg = millimeters of mercury; SE = spherical equivalent in diopters; DM = Diabetes Mellitus; CRT = central retinal thickness; CFT = central foveal thickness; ^a^ = *p*-value obtained using Mann–Whitney test; ^b^ = *p*-value obtained using chi-square test; ^c^ = *p*-value obtained using *t*-test.

**Table 3:** Overall characterization of the sample according to study group.

	Early AMD (*n* = 2)	Intermediate AMD (*n* = 16)	Atrophic AMD (*n* = 6)	Neovascular AMD (*n* = 14)	*p-*Value
Age (years) Mean (SD)	84.0 (8.5)	81.4 (3.3)	81.0 (6.5)	85.2 (5.6)	0.164 ^a^
Sex M/F *n* (%)	2 (100) 0 (0)	4 (25) 12 (75)	8 (57.1) 6 (42.9)	8 (57.1) 6 (42.9)	n/a
Study eye RE/LE *n* (%)	0 (0) 2 (100)	8 (50) 8 (50)	7 (50) 7 (50)	7 (50) 7 (50)	n/a
BCVA Mean (SD)	77 (9.9)	74.4 (8.8)	44.7 (14.7)	44.8 (23.3)	<0.001 ^a^
IOP (mmHg) Mean (SD)	13.5 (0.7)	14.4 (2.6)	15.3 (1.5)	14.6 (3.5)	0.547 ^b^
SE Mean (SD)	0.87 (0.5)	0.19 (0.9)	0.17 (0.7)	0.27 (0.8)	0.650 ^b^
Hypertension Yes/no n (%)	1 (50) 1 (50)	10 (62.5) 6 (37.5)	4 (66.7) 2 (33.3)	9 (64.3) 5 (35.7)	n/a
DM Yes/no *n* (%)	0 (0) 2 (100)	8 (50) 8 (50)	2 (33.3) 4 (66.7)	4 (28.6) 10 (71.4)	n/a
CRT Mean (SD)	255.5 (36.1)	284.6 (38.4)	222.3 (51.3)	332.9 (132.7)	0.059 ^b^
CFT Mean (SD)	223.0 (24)	217.0 (28.6)	172 (79.3)	217.9 (69.8)	0.354 ^a^

*n* = absolute frequency; n/a = Not Applicable; SD = standard deviation; M = male; F = female; RE = right eye; LE = left eye; % = proportion per group; BCVA = best corrected visual acuity; IOP = intraocular pressure; mmHg = millimeters of mercury; SE = spherical equivalent in diopters; DM = Diabetes Mellitus; CRT = central retinal thickness; CFT = central foveal thickness ^a^ = *p*-value obtained using one-way ANOVA test; ^b^ = *p*-value obtained using Kruskal–Wallis test.

## Data Availability

Data are contained within the article.
